# Defining Emerging Roles for NF-κB in Antivirus Responses: Revisiting the *Interferon-β* Enhanceosome Paradigm

**DOI:** 10.1371/journal.ppat.1002165

**Published:** 2011-10-13

**Authors:** Siddharth Balachandran, Amer A. Beg

**Affiliations:** 1 Immune Cell Development and Host Defense Program, Fox Chase Cancer Center, Philadelphia, Pennsylvania, United States of America; 2 Department of Immunology, H. Lee Moffitt Cancer Center, Tampa, Florida, United States of America; The Fox Chase Cancer Center, United States of America

## Introduction

Classic studies over the last two decades have made virus-induced activation of the mammalian *interferon-β* (*ifnβ*) gene a prototype of eukaryotic gene regulation [1]–[6]. Indeed, the compact ∼50 base-pair enhancer region upstream of the *ifnβ* transcription start site is amongst the best-studied stretches of mammalian DNA, and its function in regulation of *ifnβ* expression is considered a paradigm of stimulus-activated mammalian gene regulation.

In a widely accepted model, RNA virus infection of most cell types triggers the activation of three classes of transcription factor—interferon regulatory factors (IRFs)-3/7, NF-κB, and ATF-2/c-Jun—downstream of the RIG-I-like receptor (RLR) family of viral RNA sensors [7]–[9]. These transcription factors bind well-defined adjacent sites in the *ifnβ* enhancer to nucleate formation of an “enhanceosome”. The nascent enhanceosome then recruits chromatin-modifying enzymes and general transcription factors to initiate transcription of *ifnβ* and launch the type I IFN antiviral innate immune response [1], [2], [10]. Implicit in the inherently cooperative nature of enhanceosome formation is the supposition that IRFs-3/7, NF-κB, and ATF-2/c-Jun are all perhaps equally necessary for virus-driven *ifnβ* expression. Recent findings from our laboratories and other groups, however, suggest an alternate view of NF-κB function in antivirus responses: that NF-κB is indeed required for *ifnβ* expression, but only *before* (and very early after) infection. As the infection unfolds, NF-κB is no longer necessary for *ifnβ* induction, and instead takes on a more general role in the expression of non-IFN innate immune and pro-inflammatory genes; meanwhile, IRFs-3/7 inherit *ifnβ* expression to propel the type I IFN antiviral system. In this article, we update the enhanceosome paradigm by proposing temporally distinct functions for NF-κB in the RLR-triggered innate immune response.

## Unexpected Results from NF-κB Gene-Targeted Mice

Given that IRFs-3/7, NF-κB, and ATF-2/c-Jun assemble on the *ifnβ* enhancer, it was expected that all three factors would be critical for virus-triggered induction of *ifnβ*. In line with this expectation, studies using mice deficient in IRF-3 and/or IRF-7 have convincingly shown essential roles for these IRFs in production of IFN-β and other type I IFNs [11]–[13]. We were therefore surprised to discover that cells from mice genetically deficient in key NF-κB subunits (such as RelA, c-Rel, or p50) were mostly normal in their ability to activate *ifnβ* expression after virus infection [14]. Indeed, cells lacking virtually all detectable RLR-triggered NF-κB activity continued to support robust virus-induced *ifnβ* expression [14], [15]. Thus, while NF-κB *is* activated by virus infection and *does* associate with the *ifnβ* enhancer, it does not appear to be *required* for subsequent transcription of *ifnβ*. These findings raise two key questions: (**1**) what is the function of the NF-κB site in the *ifnβ* promoter, and (**2**) what is the function of NF-κB in virus-triggered innate immune responses, if not to activate *ifnβ*?

## Function of NF-κB before Infection: Maintenance of Basal *ifnβ* Activity

Recent work has begun to provide answers to both these questions. Using an *in silico* approach to analyze cells deficient in RelA (the primary transactivating component of virus-induced NF-κB), we have found that NF-κB controls expression of several IFN-dependent innate immune pathways by, unexpectedly, maintaining *constitutive* expression of *ifnβ* in uninfected cells [16].

It has long been known that constitutive low-level expression of *ifnβ* is necessary for maintenance of an IFN-β autocrine signal that keeps the uninfected cell in a primed state of antiviral readiness [17], [18]. Since the type I IFN antiviral system is dependent on feed-forward signal amplification, even small differences in basal gene expression translate into major downstream deficiencies. We have found that in the absence of RelA, basal expression of *ifnβ* is reduced, and autocrine IFN-β signaling is compromised. Consequently, there is a delay in the induction of *ifnβ* after infection, and, later, severe defects in the activation of the type I IFN response [14], [16], [19]. This tardiness in type I IFN feed-forward signaling has negative consequences for host antiviral immunity: RelA-deficient embryo fibroblasts are very susceptible to interferon-sensitive RNA viruses such as vesicular stomatitis virus (*Rhabdoviridae*), Newcastle disease virus, and Sendai virus (both *Paramyxoviridae*), despite producing copious amounts of IFN-β later during the course of infection [16], [19]. In these cells, diminished IFN-β expression prior to infection (and early after infection, see below) allows the virus a head start, and even though IFN-β production eventually catches up to (and even exceeds) wild-type levels, the temporal advantage conferred to the actively replicating RNA viruses during an acute infection ultimately proves insurmountable [16], [19]. These findings highlight the importance of timely IFN-β production (rather than the maximal amount produced) in innate immunity to an acute RNA virus infection.

The precise mechanism that generates constitutive NF-κB activity is currently not known. We have found that NF-κB cycles robustly through the nuclei of uninfected primary cells in an IKK-β-dependent manner, and IKK-β-deficient cells are also defective in autocrine IFN-β-mediated basal interferon-stimulated gene expression [16]. Our preliminary findings suggest that neither tumor necrosis factor-α nor Toll-like receptors (TLRs) lie upstream of IKK-β as a source of constitutive NF-κB [16].

## Function of NF-κB Early in Infection: Role in *ifnβ* Induction

In addition to controlling constitutive *ifnβ* expression, NF-κB is also the earliest-arriving *virus-activated* enhanceosome component, appearing on the *ifnβ* enhancer within 2 hours of virus infection (and approximately 2 and 4 hours ahead of ATF-2 and IRF-3, respectively) [20]. Recent elegant experiments from the Thanos laboratory show that NF-κB, despite being found in rate-limiting amounts in the cell, manages to gain such rapid access to the *ifnβ* enhancer via a novel process of inter-chromosomal transfer from putative NF-κB “receptor centers” [21]. In their model, specialized genomic loci containing readily accessible NF-κB binding sites serve as temporary receptors for incoming nuclear NF-κB, following which NF-κB is shuttled to either of two *ifnβ* loci to initiate monoallelic *ifnβ* expression. Later in an infection, feed-forward production of IRF-7 drives bi-allelic *ifnβ* expression to accelerate the type I IFN response [21].

Consistent with this model, we have also found that NF-κB has a key role in early virus-induced *ifnβ* expression [19]. This early requirement for NF-κB may stem from how the co-activator CBP/p300 is recruited to the *ifnβ* locus: an ∼30 amino-acid region within the NF-κB RelA subunit (termed the “synergism domain”) has been demonstrated to be essential for the initial capture and stabilization of CBP/p300 at the enhanceosome [22]. Although IRFs and c-Jun can independently associate with CBP/p300, the ability to *synergize* with other enhanceosome components to anchor CBP/p300 and bridge the enhanceosome to the RNA polymerase II transcriptional machinery appears to be unique to the NF-κB RelA subunit [22]–[24]. Once CBP/p300 is at the *ifnβ* enhancer (3–4 hours post infection [20]), IRFs are already robustly activated and capable of binding CBP/p300 to drive *ifnβ* transcription without further requirement for NF-κB. Indeed, IRF-3 can form a stable complex with CBP/p300 in the absence of other enhanceosome components [25], [26], and data suggest that IRF-3′s transcriptional activity can almost entirely be accounted for by its ability to capture CBP/p300 [27]. Collectively, these findings allow us to propose a model in which, early in infection, low levels of individual enhanceosome components cooperate to tether CBP/p300 to the *ifnβ* locus in a manner crucially dependent on NF-κB RelA. Later in infection (when activated IRF-3 dimers are found in larger amounts) IRF-3 can perform this function by itself, and the requirement for NF-κB is obviated. It is very likely that a similar IRF-3-dependent mechanism also accounts for *ifnβ* expression in the complete absence of NF-κB RelA [14], [19].

## Function of NF-κB Later in Infection: Regulating Pro-Inflammatory and Anti-Necroptotic Gene Expression

Once IRFs have been activated, NF-κB appears to be unnecessary for *ifnβ* expression, and instead switches to regulating a distinct set of genes that comprise roughly 25% of all RLR targets [16]. The NF-κB-dependent subset of the RLR transcriptome is especially enriched for genes encoding (**1**) chemokines, chemokine signaling, and adhesion molecules, (**2**) matrix metalloproteinases and allied proteases involved in remodeling the extracellular matrix, and (**3**) proteins involved in antigen processing and presentation, including a large number of classical and non-classical major histocompatibility class I molecules. In addition, RelA is also weakly activated by IFN-β itself [16], [28], and is required for induction of a small subset (<5%) of interferon-stimulated genes (most notably those encoding chemokines CxCl11 and Ccl3) [16]. Finally, RelA-deficient cells treated with the virus mimetic poly(I:C) are very susceptible to a novel form of cell death termed necroptosis [29], [30], indicating that RelA might also transcriptionally control a cell survival program to prolong pro-inflammatory gene expression from the infected cell [16], [31]. Collectively, these findings show that the NF-κB arm of the type I IFN antiviral response is focused primarily on generating pro-inflammatory and pro-survival signals, rather than on activating cell-intrinsic antiviral effectors (or on feed-forward amplification of IFN signaling itself).

## Conclusions

**Figure 1:**
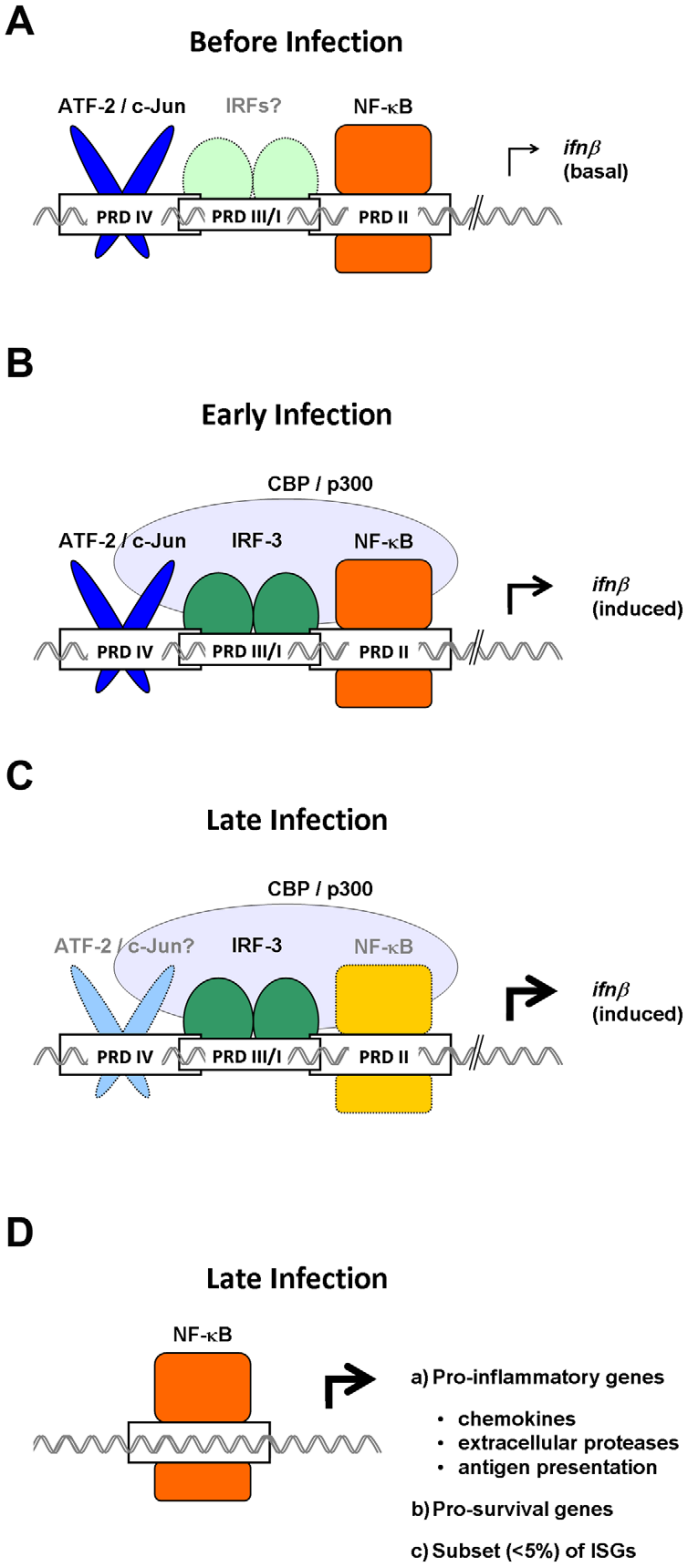
Temporally distinct roles for NF-κB in antivirus innate immune responses. (A) In uninfected cells, NF-κB cycles robustly through the nucleus to maintain constitutive expression of basal *ifnβ* and sustain sutocrine IFN-β signaling. (B) Early in an infection, NF-κB cooperates with ATF-2/c-Jun and IRF-3 to recruit the transcription co-activator CBP/p300 to the *ifnβ* enhancer. (C) Later in an infection, IRF-3/7 powers expression of *ifnβ*, and NF-κB is rendered redundant in the *ifnβ* enhanceosome. (D) NF-κB then switches to regulating a distinct subset of non-IFN genes, including those involved in inflammation and cell survival. The relative importance of each transcription factor in driving gene expression during a particular stage of the immune response is indicated by the intensity of its color, with darker shades representing essential functions, and lighter shades indicating reduced roles.

We propose here an updated view of NF-κB's overall function in the innate antivirus response, in which NF-κB has a crucial constitutive (and early) role in *ifnβ* expression followed by an equally important and potentially more general later role in regulating expression of genes involved in recruitment and activation of the adaptive immune response. Interestingly, other groups have demonstrated that c-Jun also participates in maintenance of autocrine IFN-β, while IRF-3 and IRF-7 may not [32], [33]. Taken together, these findings support the idea that NF-κB and c-Jun sustain basal/early *ifnβ* expression, while IRF-3 and IRF-7 instead dominate IFN-β production following virus infection (Figure 1). Important areas for future investigation include: (**1**) the source of constitutive NF-κB activity; (**2**) the role of other IRFs (for example, IRF-1) in constitutive *ifnβ* expression; and (**3**) evaluation of cell type-specific roles for different NF-κB subunits in anti-virus responses *in vivo*. For example, the key type I IFN producing plasmacytoid dendritic cells utilize TLRs, rather than RLRs, to activate *ifnβ* [34]. Is the requirement for—and subunit composition of—NF-κB in these cells the same as it is in cells that deploy a RLR-driven IFN response? Despite over two decades of investigation, the regulation of *ifnβ* expression continues to throw up surprises, and more unanticipated findings are likely forthcoming.
